# A Preliminary Investigation of Social Network Analysis Applied to Dairy Cow Behavior in Automatic Milking System Environments

**DOI:** 10.3390/ani11051229

**Published:** 2021-04-24

**Authors:** Liliana Fadul-Pacheco, Michael Liou, Douglas J. Reinemann, Victor E. Cabrera

**Affiliations:** 1 Department of Animal and Dairy Sciences, University of Wisconsin-Madison, Madison, WI 53706, USA; vcabrera@wisc.edu; 2 Department of Statistical Science, University of Wisconsin-Madison, Madison, WI 53706, USA; myliou@wisc.edu; 3 Biological and Systems Engineering Department, University of Wisconsin-Madison, Madison, WI 53706, USA; doug.reinemann@wisc.edu

**Keywords:** cow management, cow behavior, affinities

## Abstract

**Simple Summary:**

Cows are social animals, therefore understanding the ways that they interact can help improve their management and welfare. We used social network analysis (SNA) to data on voluntary cow movement through a sort gate in an automatic milking system to identify pairs of cows that repeatedly passed through a sort gate in close succession (affinity pairs). Results from this exploratory study showed that when cows were separated from their affinity-pair cow the day-day variability in milk production increased by a factor of 3, a possible indicator of stress. The results of this exploratory study suggest that SNA could be used as a tool to better understand the social dynamics of dairy cows and inform group and regrouping process to produce positive outcomes.

**Abstract:**

We have applied social network analysis (SNA) to data on voluntary cow movement through a sort gate in an automatic milking system to identify pairs of cows that repeatedly passed through a sort gate in close succession (affinity pairs). The SNA was applied to social groups defined by four pens on a dairy farm, each served by an automatic milking system (AMS). Each pen was equipped with an automatic sorting gate that identified when cows voluntarily moved from the resting area to either milking or feeding areas. The aim of this study was two-fold: to determine if SNA could identify affinity pairs and to determine if milk production was affected when affinity pairs where broken. Cow traffic and milking performance data from a commercial guided-flow AMS dairy farm were used. Average number of milked cows was 214 ± 34, distributed in four AMS over 1 year. The SNA was able to identify clear affinity pairs and showed when these pairings were formed and broken as cows entered and left the social group (pen). The trend in all four pens was toward higher-than-expected milk production during periods of affinity. Moreover, we found that when affinities were broken (separation of cow pairs) the day-to-day variability in milk production was three times higher than for cows in an affinity pair. The results of this exploratory study suggest that SNA could be potentially used as a tool to reduce milk yield variation and better understand the social dynamics of dairy cows supporting management and welfare decisions.

## 1. Introduction

Dairy cows are social animals, therefore understanding the ways that they interact has been identified as a valuable tool to improve management decisions affecting their welfare [1] and to control disease transmission [2,3]. Social interactions in domestic animals can be affiliative (i.e., positive interactions) or agonistic (i.e., aggressive or competitive interactions), where the former may contribute to social support and reduce distress behavior [4,5]. Affiliative relationships are an important component of the “social buffering” theory which postulates the importance of social support to provide beneficial effects to the individual involved and may improve welfare by reducing stress [6,7].

Living in groups provides benefits that can increase dairy cow welfare and fitness [1]. Moving animals between groups within the herd is a common practice to create homogenous groups according to different characteristics (i.e., age, lactation, milk production, health status, etc.). Yet studies have reported that regrouping cows can have negative effects on welfare and milk production after regrouping [8,9,10]. The negative effects of regrouping cattle include decrease in lying time, increase in time standing, increase in agonistic interactions and changes in feeding behavior (i.e., decrease in rumination and time feeding) [8,9,10,11]. Also, an increase in fecal cortisol concentration (used as a physiological indicator of stress) in primiparous cows when they were moved without their partners [12]. Milk production could be affected after regrouping cows, probably due to the competition for access to food and lying spaces [8,10]. Further, Mazer et al. [12] measured fecal cortisol, a physiological indicator of stress, when regrouping cows alone or with a familiar partner. They found that cortisol concentration was higher in first lactation cows than in multiparous cows when they were regrouped without their partner, but there were no differences in cortisol levels between parities when the cows where regrouped with their familiar partner. However, in cows, the decrease in milk production (around 3.7/kg per cow and day) has been seen only on the day after mixing [10]. In heifers, reductions of milk production up to 96.5% had been reported, during 1 to 2 weeks after regrouping [13]. Even if it is for a short period, it is still of great importance from the economic and welfare standpoints.

Social network analysis (SNA) explores patterns of complex interactions in communities and provides a description of the structure of social relationships [14]. Previous studies with SNA in cattle have been done using localization tags [15,16]. Research has shown that cows with a high degree of familiarity are eager to stay closer to each other, while unfamiliar cows tend to evade each other, and all social relationships tend to reduce among them [17]. The concept of spatial proximity as a way to evaluate preferential affinities is widely accepted [18]. The automatic milking systems (AMS) environments provide a real-time and readily accessible measure of social relationships. The AMS record when cows pass through a sorting gate when moving from resting to feeding or milking areas, and when cows are presented to the AMS to be milked. These time series data can be used to assess cow relationships as they move throughout their environment. Barns with AMS are designed to have groups of cows ranging from 50 to over 200 cows per pen depending on herd size, barn design and number of AMS. Evidence suggests that behavior of individual cows may be crucial on the efficient use of AMS [19].

A study by Gutmann et al. [17], reported that dairy cows develop and maintain dyadic relationships. It could be that these subgroups provide a stable structure to reduce social conflicts within the herd. Group dynamics depend on the characteristics of the cows that are introduced to or leave a group [20,21]. Likewise, cows with previous regrouping experience will have a faster social integration (i.e., 0–2 days with regrouping experience vs. 2–4 days without regrouping experience) [8] and develop social support (i.e., stress buffering effect) [6] to improve animal welfare [17]. Our interest was to gain a better understanding of social relationships to aid in management decisions to improve animal welfare and cow performance on farms using AMS technology. Since social behavior is the principal element of farm animal welfare [6] and social behavior in cattle is described by the creation and maintenance of cohesive social groups [17], leading to management practices more in line with the natural social behavior of the cows [3].

A steady stream of cows that present themselves for milking is required to keep the AMS fully engaged in the milking process and cow movement may be influenced by social grouping structure. We hypothesized that broken affinities (i.e., separating cows by removing one from the pen) could alter the behavior or stress level of a cow and therefore impact production. Hence, the objective of this study was to perform an exploratory analysis using SNA with cow behavior data obtained from an AMS environment to identify locational affinity of pairs of cows, as defined by a cow repeatedly passing through the selection gate in close succession to its affinity-pair cow. Cow pair locational affinity is referred hereafter as social relationships. A further analysis was performed to assess if being part of an affinity pair affected milk production.

## 2. Materials and Methods

### 2.1. Animals and Housing

This study was done with data from a commercial dairy farm located in Wisconsin, United States, during a 12-month period (4 February 2019 to 4 February 2020). Cows were confined (i.e., zero-grazing) and the farm practiced year-round calving. A consistent diet consisting of a partial mixed ration (PMR) composed of haylage, corn silage, shell corn and protein mix was supplied to all the pens throughout the year. The PMR was formulated to cover the basic requirements of the cows in the herd. In addition to the PMR, cows receive corn gluten pellets when they are milked by the robot. The amount of pellet feed depends on the lactation, days in milk (DIM) and milk production. The farm has four pens with one Delaval (DeLaval International AB, Tumba, Sweden) AMS per pen. On average the farm had 214 ±34 milking cows, averaging about 54 cows per AMS during the study period. Each pen was fitted with a preselection sorting gate (SG) that allowed cows voluntarily moving from the resting area to be directed to either a holding area for the AMS or to the feeding area. After milking, cows were directed to the feeding area or to a catch pen where human examination or intervention could be applied. Cows then moved from feeding areas back to the resting areas via one-way gates. Cows passed through the selection gate about 10 times per day and were diverted to the AMS for milking (and then released to the feeding area) 2.7 times per day. The management plan of the farm was to group cows according to lactation number, however, the practicality of keeping pens full and limitations on the pool of available cows meant that this grouping plan count not be strictly adhered to. Descriptive statistics of the pens are presented in Table 1.

### 2.2. Data

Data on cow traffic and milk performance were made available through the Dairy Brain project at the University of Wisconsin-Madison [22]. Data on cow traffic provided the time of the day that each cow passed through the SG in each pen. Milk performance data from the AMS included milk yield, the time each cow was milked, lactation number and DIM for each cow. In addition, we identified 142 lactation numbers that were incorrectly recorded, and the data were manually corrected. We treated the missing data as missing at random because there was a small amount of missing data without a clear pattern of occurrence. Missing data was applied equally to all cows in the pens and therefore not expected to introduce bias to the identification of affinity pairs.

### 2.3. Scoring and Network Representation

We considered three different approaches to characterize affinities: (1) All cows that went through the SG in a 15 min window were considered to have a uniform relationship with one another within the same window; (2) A continuous scoring method with affinity weighting applied inversely to the time interval that the cows passed through the SG; and (3) Lag sequence decreasing score: An exponentially decreasing score assigned according to the ordered sequence of cows passing through the SG, up to four consecutive cows. There were no major differences in the affinity pairs or number of pairs identified across these three approaches. We chose to use the lag sequence method to avoid the use of a pre-defined time window or time weighting [15]. Waters and Fewell [23] suggest that different time frames can be used to examine a social network and the question of the optimal time window merits further investigation.

**Figure A1:**
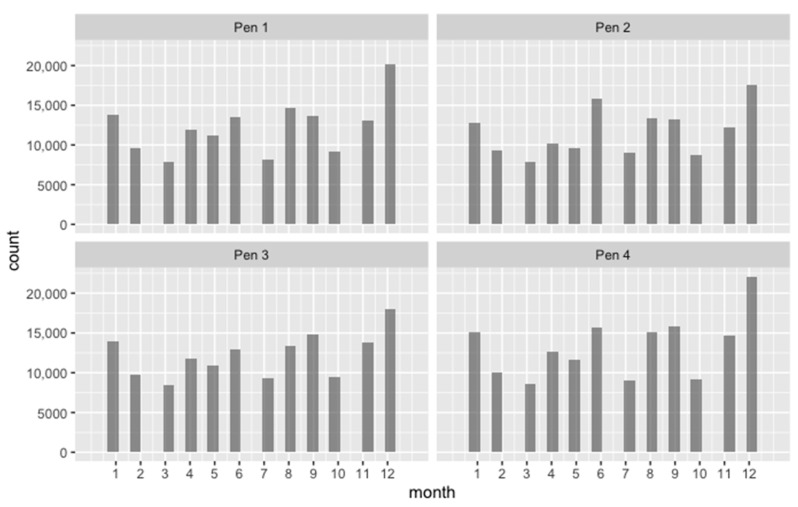
Count of the gate passage per month by pen.

In addition, under husbandry conditions, herd structure is dynamic primarily due to management decisions, and some relatively small number of cows could enter or leave the pen for different reasons (e.g., start or end of lactation, sold or health issues) however, in general, the majority of the cows, 40 cows on average per pen, were present on the pens from 181 to 270 days of the year (Table 2). Data for cows with less than 30 days residency time in the pens were not considered. The affinity pair scores were normalized by the total time each cow spent in the pen during the study period (Table 2). Furthermore, since the net traffic information from the SG varies across the year (Figure A1), a second score normalization was done to characterize how cow affinities and cow networks change over time. Months within one standard deviation above the mean of gate passages across all pens (i.e., December in all pens, June in pens 2 and 4 and September in pen 4) were normalized such that observations during these months weighted up to 2.8 times less than months with the lowest count of gate passages (e.g., March in all pens and July in pen 1) (Figure A1). Months with low count of gate passages have no relation to the social network of the cows, or bias against the frequency in which the cows pass through the gates, however the AMS logged traffic for certain months differ throughout the year, thus this adjustment was necessary. We assumed that this normalization, that accounts for farm management, could change the interpretation made for relationships between the cows, but rather, simply makes the social affinity scores between cows more comparable across months.

### 2.4. Social Network

Social networks were represented by weighted (i.e., describing the number of interactions), undirected (relationships were symmetric, i.e., neither cow was considered dominant in the paring) graphs, and summarized as a weighted adjacency matrix with each row and column denoting a single cow. Nonzero entries were weighted relationship scores for each edge (connection) between nodes (cows), as described in the previous section, while zero entries indicate no relationship between the cows and thus no edge [14].

The SNA metrics were the first step to explore the scores from each network by pen, as they asses the different roles of the different individuals in the network [14,24]. We included diameter, density, degree centrality of a node and node centrality. The diameter is the shortest path length in the network, and the average path length is the average number of contacts along the short path between all the pairs of the network (i.e., how close they are to each other) [2]. Density measures the proportion of contacts between the node, and its value ranges between 0 and 1, where 0 mean absence of a network or no contacts and 1 is a fully connected network [2,25]. The degree centrality of a node measures the connectivity among nodes (i.e., higher values mean more contacts) [2,14]. The node centrality measures the shortest paths that pass through the node, indicating how likely important is a node to connect largely independent networks [14,25].

### 2.5. Maximum Social Affinity Pairs

Periods of affinity were identified using SNA to find pairs of cows with the highest affinity over the entire study period. Periods of time in which the cow’s behavior was not associated with its cow pairs were hypothesized to be periods of no, or lower social affinity. We considered the relationship to be symmetric implying that neither cow was dominant in the pairing. This may be a limitation of our study as we did not investigate whether the relationships were truly symmetric and there could have been a dominant cow in the pairing. Another limitation of our study is that we identified only one pairing.

These limitations are illustrated by a consideration of three cows, in which cow A is in the pen for the entire period of the study, and cow B and C for two non-overlapping periods. The highest pairing score for cow B is cow A, but it may be possible that cow A’s highest score is with cow C. Although each cow may have more than one affinity cow this complicates the analysis of milk production. This is a statistical problem that we do not have the tools to solve right now. For example, if we consider a star shaped graph, in which a single cow is the only common affinity between many others, we would be unable to define a period of time for comparison of affinity and non-affinity. Ideally, for a single cow, we would look at the time period that the cow is with all its affinities and compare that time period against a different time period in which that affinities of that cow were broken, and still be in the same parity and days in milk. However, this period is not straightforward to define if the cows are moving in and out of the pens. Thus, trying to analyze groups of cows will likely introduce bias in unknown directions depending on each cow. We avoid this by only looking at symmetric cow pairs, which define precise periods of the cows when they were together with their pair (affinity period) and when they were without their pair (period where the affinity was broken). Although an approximation to social affinity, we think this to be the cleanest way to study this problem statistically.

Finally, we may consider an additional stability criterion for the associations made. Stability of matched pairs would mean that affinity preference cannot be improved with better scores by switching partners with anyone else. That is, no cow can switch matched cow pair without at least one cow getting a lower scoring cow. Although most interpretable, stable, exclusive and symmetric pairings are not always possible. See the stable roommate matching problem for more detail [26]. We considered 3 different algorithms in a stepwise manner for pair generation: (1) If exclusivity of cow pairs is not considered, we simply take a greedy approach in assigning the top scoring partner for each cow as their cow pair. (2) If cow pairs are deemed exclusive, but a stable matching constraint (no switching of cow pair would result in a better matching for both pairs) is placed on the pairs generated, we are in the scope of the “stable roommates” problem and can use Irving’s algorithm [26]. (3) A stable matching is not always possible, and thus we default to a weighted blossom matching algorithm, in which a maximal pair matching is found based on the edge weights. Pairs are determined at the pen level, and pairs with low scores (i.e., score = 0) are excluded from the analysis.

### 2.6. Statistical Analysis of Daily Milk Yield

**Figure 1:**
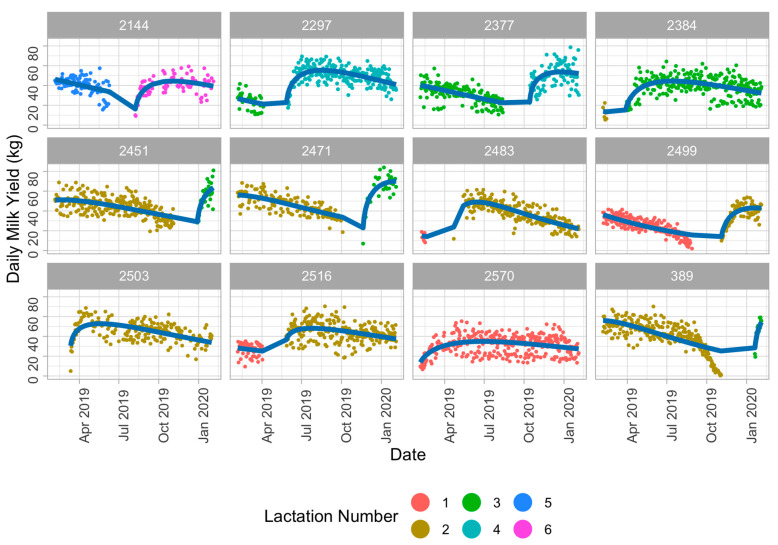
Examples of the expected and actual milk production data. The expected values are determined from the nonlinear, Wood’s curve mixed model with random effects of lactation number nested within cow. Here we illustrate that the trends in actual milk production correspond to the expected values, which account for lactation number and stage of lactation (blue line).

To assess the effect of social affinities on daily milk yield, we used the following framework: For each cow, we modeled an expected daily milk production curve using data available during the 1-year study period. This curve serves as a basis of comparison, in order to understand if a particular cow is over-producing or under-producing adjusted for their lactation and stage of lactation. We liken this sort of analysis to a field of causal inference in statistics called synthetic controls [27], in which we use the entire population of cows to create a “synthetic cow” that is the idealized version of the cow we make comparisons for. Given this synthetic cow, accounting for observed variables, is independent from determining the periods of social affinity comparison. To model the expected lactation curves, we used a nonlinear, mixed effects model with random effects of lactation nested inside each cow. We used the Wood’s lactation curve model [28] as the functional form of the nonlinear regression (1).

(1) $$Y_{ti}=\alpha_{i}t^{\beta_{\iota }}exp\left(-\gamma_{i}t\right)$$

where *Y_ti_* is the milk yield (kg) at a day *t* for cow *i*, *α*, *β* and *γ* are parameters that determine the lactation curve’s shape. We simplified the random effect structure of the model with guidance from the data, and the variance explained by the groupings for each parameter. The final model was a Wood’s lactation curve fit with varying parameters *α*, *β* and *γ* for every cow and lactation combination. Examples of the Wood’s lactation curve model and individual cow production are illustrated in Figure 1.

The deviation from the Wood curves was fitted to evaluate the performance of the cows when they were together with their pair (affinity period) and when they were without their pair (period where the affinity was broken or separating pairs of cows by removing one from the pen), summarized by the difference in the area between observed and expected lactation curves. We erred on the side of overfitting the modeled curve for each cow by means of a more complex random effect structure. This likely provides a more conservative estimates of the differences between observed and expected values under stress conditions for the cow.

**Figure A2:**
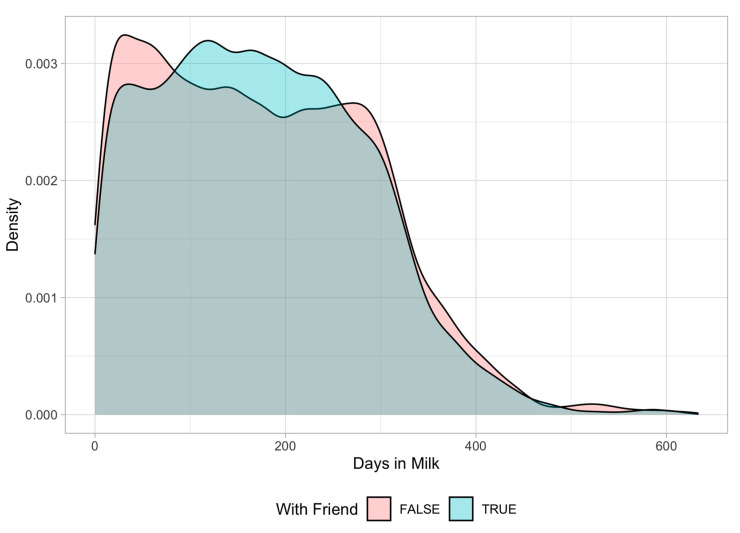
Overlap of periods of social affinity as a function of days in milk. The density of observations in periods of social affinity (green) and non-social affinity (red) have similar distributions.

As the daily variability in milk production is much larger during early lactation, we also verified that there was no spurious confounding correlation with the periods of social affinity and days in milk. The distribution of days in milk between periods of social affinity and non-social affinity was similar (Figure A2), thus supporting a valid comparison.

We hypothesized that during periods of animal’s social affinity, milk production levels would be higher than during periods when no affinity was observed or when social affinities were broken. A paired t-test analysis was performed for each pen for the summarized difference in milk production (kg/day) for periods of social affinity versus periods without social affinity, in which cows were paired based on the social network scoring. All the data preparation and analysis were performed with the R statistical software version 3.6.2 [29]. Specifically, we used the packages: “igraph” [30]; “matrix” [31]; “ggraph” [32] and “lme4” [33].

## 3. Results

### 3.1. Social Network

**Figure 2:**
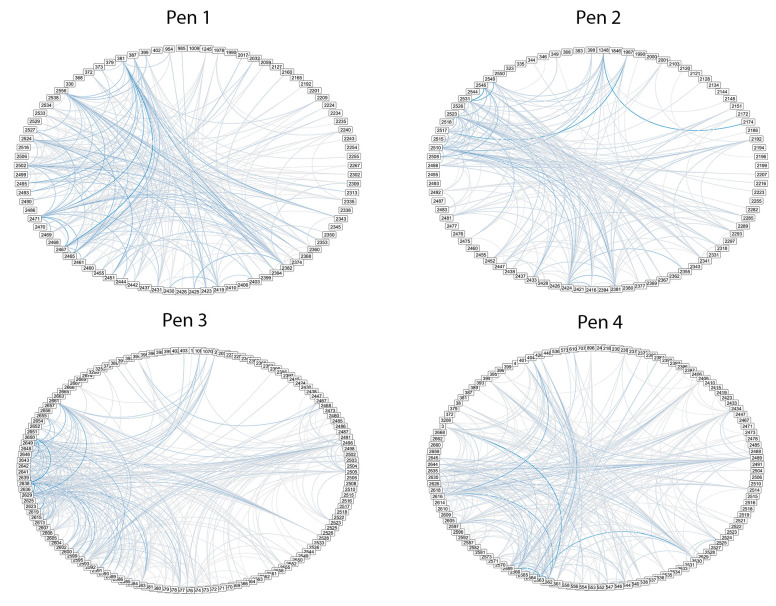
Network representation of pens’ social networks scored with the lag sequence decreasing score where the top 10% of network edge weights are shown. Each node is an individual cow (cow numbers forming the outer circle), and each edge (blue lines inside the circle) is a connection between cows with darker lines indicate strength of the relationship between the cows. Only the strongest 10% of the edges are shown to avoid oversaturation of lines.

The top 10% of the network edge weights are illustrated in Figure 2. Although all the pens had dense networks, the density values were greater than 0.70 for pen 1 and 2 and 0.46 and 0.52 for pens 3 and 4, respectively. The degree centrality values (i.e., number of affinities that a cow has) confirm that the networks in all four pens are fully connected (Table 3). The number of cows with the greater number of connections were found in pens 1 and 2 (Table 3). This result is highlighted by the number of cows and the time spent on pens 1 and 2 as compared to pens 3 and 4 (Table 2). In addition, results show that there are at least one or two cows that remain within the top five relationships from month to month.

### 3.2. Maximum social Affinity Pairs on Daily Milk Production

**Figure 3:**
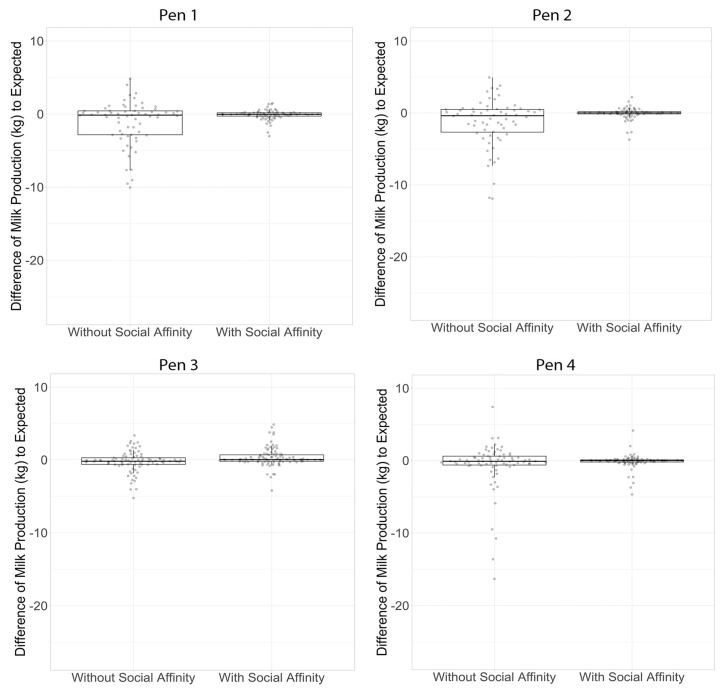
Difference of milk production by pen between cows during period with social affinity and periods without social affinity.

Our primary finding was that the separation of cows from their pair (period where the affinity was broken), lead to an increase in the day-to-day SD of milk production from 0.80 kg/day for periods of affinity to 2.62 kg/day for periods of no affinity, or more than a 3-fold increase (Table 4). Furthermore, we found that in one out of the four pens, milk production was significantly lower than the Wood’s curve expected value during periods of non- affinity (Pen 3: 0.66 kg/day per cow, *p*-value = 0.03). Although the differences in milk production were non-significant in the other pens, milk production was numerically lower, and milk production variability was higher in periods where social affinities were broken compared with periods of social affinities (Table 4 and Figure 3). This increased variability in milk production also likely contributed to the lack of finding significance for the milk production effect.

## 4. Discussion

### 4.1. Social Network

We used sequential gate passes as our proxy for social interaction between cows. This method does not include any information about how cows interact while in resting, milking or feeding areas and cannot distinguish between positive and negative social interaction. These was not part of this exploratory study but should be possibly included in further research. However, changes in the variability of milk production provides some evidence that sequential gate passing, and SNA more generally, are interesting avenues of exploration. Gygax et al. [15] suggested that when movement is not restricted animals have more independence to choose their partners. In AMS situations cow movement is voluntary and the presence of a sort gate provides data on at least one aspect of behavior that may indicate relationship.

The social network of dairy cows had been reported to be dynamic in time and affected by the characteristics of specific cows that are introduced to, or leave the group [3,20,21,34]. Foris et al. [21] reported that cows had consistent social behavior characteristics over a 6-month period and other studies have reported non-random selection of cow pairing [15,17]. These results are an indicator of the stability, and perhaps importance of cow-cow relationships.

Our density results from pens 3 and 4 are comparable to those reported by Gygax et al. [15] (i.e., 0 to 0.50) as well as with the results reported by Foris et al. [21] (i.e., 0.28 to 0.81). The degree centrality values (i.e., number of affiliations that a cow has) confirm that these are fully connected networks (Table 3). Fully connected networks for dairy cows had been also reported by other studies [2,20]. The higher density of the networks in pens 3 and 4 as compared to pens 1 and 2 in our study could be explained by the higher number of cows (i.e., number of nodes) during the studied period (Table 3) and also by the individual personality of the cows [20,21]. Individual cow traits have been related to the dominance position in a social network [34,35].

### 4.2. Effects of Maximum Social Affinity Pairs on Daily Milk Production

Milk production could be negatively affected after regrouping cows, probably due to the competition for access to food and lying spaces [8,10]. Therefore, the day-to-day variability on milk production could be an indication of social stress. The significant reduction in milk production found in pen 3 and not the other pens could be because a higher percentage of cows in pen 3 were first lactation cows than in other pens and an indication that the social dynamics of first lactation cows is different than for multiparous cows. Mazer et al. [12] found that primiparous cows had higher cortisol concentration when they were regrouped compared to multiparous, showing that first lactation cows are potentially at a higher risk of experiencing stress when the affinities are broken. Differences among pens could also been explained by the position, hierarchy, influence and/or identity of each individual in the network [20,24,36], since the group dynamics depend on the characteristics and the personality of the cow that is introduced to or leaves the group [20,21,34].

Our results do suggest that cows separated from their affinity pair had greater day-day variability in milk production. The increase in day-day variability in milk production in all pens when affinities were broken suggests a negative effect of losing affinity, or conversely, the positive effect of affinity. However, in this study it was not possible to differentiate between positive or agonistic interactions from the SG data. Furthermore, a definite interpretation between affiliative or agonistic interactions remains elusive [37,38]. We hypothesize that moving cow pairs together into new pens could reduce daily variation and loss in milk production that has been documented when cows are moved individually. Likewise, Gutmann et al. [17], found that cows maintain beneficial dyadic relationships and suggested that maintaining cows with their mates can contribute to cow welfare, as it helps maintain a stable social structure in the herd.

Although spatial proximity as a measure of affiliative behavior is well recognized [18], we acknowledge that the SG data we used may not be an indication of positive affiliation between cows. Even though, it is possible that cows that go through the gate sequentially within a short period of time may have an antagonistic relationship or are dominant cows [36]. Studies have reported that cows spent more time with particular single animals, which imply that cow affinity is a not-random event, and that cows have distinct roles in the social network of a pen [15,17,20]. The results of Gygax et al. [15] showed that social networks are tightly knit for attachment relationships and less dense for avoidance relationships and were denser for median distance than for synchronicity.

The question of whether gate-passing behavior can be used as a useful indicator of either affiliative or agonistic behaviors requires further investigation, however, this exploratory analysis has inspired us to further validate and investigate the method. Our results suggest that the SNA is a useful tool to evaluate cow behavior as our exploratory study found that cow affinities can impact milk production and milk production variability. To address the limitations of our study we suggest that the use of SG data could be validated and perhaps complemented with other types of data as GPS tracking systems or image analysis to better understand interactions among cows throughout time and space. More detail on the spatial proximity at all the locations (feeding, resting, milking) would help understand cows’ behavior and interactions. Additionally, in this exploratory study only pairs of cows where evaluated. A broader evaluation of relationships is also merited.

## 5. Conclusions

A better understanding of social relationships could result in decision support tools to improve AMS performance, avoid loss of milk yield, and improve animal welfare on farms using both AMS and conventional milking technology. The results of this exploratory study suggest that cows’ social relationships can impact the day-to-day variability in milk production as well as daily average milk production. These results could lead to improved strategies for formulating and reformulating groups of cows in AMS settings. Social Network Analysis may be a promising tool to better understand cow social dynamics and the resulting performance of the dairy herds. Further validation and exploration of the method is necessary.

## Figures and Tables

**Table 1:** Average and standard deviation by pen of farm characteristics for a 12-month period (February 2019–February 2020).

Item	Pen 1	Pen 2	Pen 3	Pen 4
Number of cows	55.9 ± 7.9	54.7 ± 6.1	51.1 ± 13.4	52.4 ± 9.6
Distribution of lactation numbers *	L2 (35%)	L2 (28%)	L1 (77%)	L1 (45%)
	L3 (26%)	L3 (19%)	L2 (20%)	L2 (33%)
	L4 (26%)	L1 (14%)		L3 (16%)
		L5 (14%)		
Milkings/cow/day	2.66 ± 0.68	2.67 ± 0.80	2.67 ± 0.81	2.68 ± 0.83
Milk production (kg)/cow/day	40.5 ± 13.4	41.7 ± 14.5	36.2 ± 11.5	38.8 ± 12.3
Milk production (kg)/AMS/day	2262	2282	1843	2036

* Proportion of cows in lactation: 1 (L1), 2 (L2), 3 (L3), 4 (L4) and 5 (L5).

**Table 2:** Number of cows and range of the time (day) spent by pen during the 12-month study period (February 2019–February 2020).

Pen	Days in Pen			
	30 to 90	91 to 180	181 to 270	>271
Pen 1	26	23	41	14
Pen 2	15	26	40	15
Pen 3	44	19	29	26
Pen 4	39	31	39	11

**Table 3:** Social network metrics of the four pens.

Items	Pen 1	Pen 2	Pen 3	Pen 4
Number of nodes ^1^	84	84	129	116
Number of edges ^2^	2923	2425	3781	3456
Diameter ^3^	247	279	253	279
Density ^4^	0.84	0.70	0.46	0.52
Degree ^5^				
Maximum	80	72	99	91
Minimum	2	2	2	2
Average	70	58	59	60
Number of cows with the maximum number of connections	41	30	8	19

^1^ Number of nodes: total number of cows passing through the sorting gate; ^2^ Number of edges: number of connections between cows; ^3^ Diameter: shorter path among connections in the network; ^4^ Density: proportion of contacts between nodes (i.e., cows) and range between 0 (no contacts) and 1 (fully connected network); ^5^ Degree: connectivity among nodes (i.e., cows), higher values mean more contacts.

**Table 4:** Difference between actual and expected (predicted from Wood’s curve model) milk production for periods with and without affinity.

	Difference of Actual to Expected Milk Production, kg/day				
Item	Mean		SD		Pair *t*-Test
	No Affinities	Affinities	No Affinities	Affinities	*p*-Value ^1^
Pen 1	−0.18	−0.10	3.30	1.15	1.000
Pen 2	−0.72	−0.37	3.90	1.18	1.000
Pen 3	−0.53	0.13	2.13	0.82	0.032
Pen 4	−0.29	−0.05	3.30	0.52	1.000
Overall	−0.31	−0.11	2.62	0.80	NA ^2^

^1^ Holm corrected; ^2^ Non applicable.
